# Serological diagnosis of strongyloidiasis: An evaluation of three commercial assays

**DOI:** 10.1371/journal.pntd.0012319

**Published:** 2024-07-05

**Authors:** Thomas Weitzel, Sabine Dittrich, Frank P. Mockenhaupt, Andreas K. Lindner

**Affiliations:** Charité—Universitätsmedizin Berlin, corporate member of Freie Universität Berlin and Humboldt-Universität zu Berlin, Institute of International Health, Charité Center for Global Health, Berlin, Germany; Centre for Tropical Diseases, ITALY

## Abstract

**Background:**

Strongyloidiasis is caused by a neglected nematode, manifesting as chronic intestinal infection with potentially severe manifestations. The disease is an emerging problem in non-endemic countries affecting travelers and migrants. Diagnosis of strongyloidiasis is hampered by the lack of standardization and absence of a gold standard. Since adequate direct methods to detect the motile larvae in stool samples are not widely available, other techniques such as serology have been developed.

**Methods:**

We evaluated three commercial ELISA kits (DRG Instruments, IVD Research, and Bordier Affinity Products) to detect IgG antibodies against *Strongyloides stercoralis* assays utilizing serum samples from travelers with microscopically confirmed strongyloidiasis (n = 50) and other imported helminthic infections (n = 159) as well as healthy controls (n = 50).

**Results:**

The DRG, IVD, and Bordier assays showed sensitivities of 58.0%, 64.0%, and 56.0%, respectively. Specificity values were 96.0%, 96.0%, and 92.0% in healthy controls, and 67.3%, 62.9%, and 76.7% in cases with other helminth infections, respectively. Cross-reactions were mostly observed in cases with other nematodes (37.5%, 42.5%, and 20.0%, respectively), but also in trematode (33.3%, 38.1%, and 19.0%, respectively) and in cestode infections (25.0%, 30.0%, and 32.5%, respectively).

**Conclusion:**

The study demonstrates the diagnostic limitations of serological assays to detect or exclude cases of strongyloidiasis in returning travelers, who frequently present with recent or acute infections.

## Author summary

Strongyloidiasis is a neglected nematode infection, which causes significant morbidity and mortality due its chronic course and potential of severe manifestations. The disease mostly threatens inhabitants of tropical and subtropical regions. Moreover, it has been recognized as an emerging problem in industrialized countries affecting migrants and returning travelers. The diagnosis of the intestinal helminth is challenging, since its reliable detection in stool samples requires specific methods, which are mostly not performed. Antibody detection by serological tests is a practical and commercially available diagnostic alternative, but the capabilities of such tests in non-endemic regions are not sufficiently studied. The presented study evaluated the performance of three commercial *Strongyloides* ELISA kits using serum samples from travelers with proven strongyloidiasis or various other parasitic infections. The study revealed that the sensitivity of *Strongyloides* serology in patients from non-endemic countries, who often present with early infections, might be limited and that tests frequently cross-react with other helminth infections. Serological results should therefore be interpreted cautiously and used together with parasitological methods to reduce the risk of missing this potentially severe infection.

## Introduction

*Strongyloides stercoralis* is a neglected soil-transmitted nematode causing significant morbidity and mortality [1]. Due to its capacity of autoinfection, *S*. *stercoralis* is one of the few intestinal helminths that can lead to chronic infection [2]. In immunocompromised patients, this replicative cycle can lead to high parasite loads (“hyperinfection syndrome”) and extra-intestinal dissemination with a high fatality rate [3]. Such complications threaten patients undergoing immunosuppressive therapies even decades after exposure and infection [3]. In general, strongyloidiasis is associated with poor sanitary conditions and tropical/subtropical climates, but autochthonous cases can also occur in temperate climates and in industrialized countries [4,5]. Due to a lack of systematic studies and diagnostic standards, the burden of this neglected tropical disease is underestimated, and the exact epidemiology is poorly understood [6,7].

In industrialized countries, strongyloidiasis is an emerging medical problem, mainly affecting migrants and travelers returning from endemic countries. In populations at risk, strongyloidiasis should be suspected in patients with chronic or recurrent abdominal symptoms, malabsorption or unclear eosinophilia, and ruled out before immunosuppressive therapy and solid organ transplantation (donors and recipients) [8–10]. Despite its clinical relevance and wide distribution, most physicians are not familiar with this parasite, which can lead to severe clinical consequences [11]. The diagnosis of strongyloidiasis outside specialized centers is challenging [7]. Routine concentration techniques for ova and parasites (O&P) are insensitive and *Strongyloides*-specific direct methods such as Baermann technique are often not performed. Consequently, some guidelines promote the use of serological tests, mostly ELISAs, which are commercially available and easy to perform, but not always sufficiently evaluated [7,12,13]. The present study evaluated the performance of three commercial *Strongyloides* kits using a broad panel of serum samples from patients with parasitologically proven strongyloidiasis and other parasitic infections.

## Methods

### Ethics statement

The retrospective study used anonymously coded left-over samples and did not include demographic and clinical information, which precluded identification of individual cases. An institutional ethics approval was therefore not required.

### Patients and serum samples

The study used 259 serum samples, stored at -80°C at the serum bank of the Institute of International Health in Berlin, Germany (S1 Table). Of those, 209 specimens originated from travelers attending the Institute’s outpatient clinic, which serves as a regional reference center for tropical and parasitic diseases. Positive cases were detected within routine diagnostic procedures performed in the Institute’s diagnostic laboratory. Strongyloidiasis cases were mainly returned travelers, the majority of German origin; serum samples from other parasitic infections derived from travelers and migrants. Strongyloidiasis was diagnosed using the Baermann technique. This simple method permits the visualization of the motile larvae, with subsequent morphological identification. The identification of other intestinal helminths relied on the detection of eggs (after Merthiolate-Iodine-Formalin [MIF] stool concentration) or of adult worms. *Schistosoma* spp. eggs were detected in stool (after MIF stool concentration), in urine (after filtration) or in tissue biopsies. Eggs of *Paragonimus* spp. were identified in sputum after centrifugation technique. Detection methods for microfilariae included blood filtration and skin snip technique. Cases of echinococcosis and cysticercosis were diagnosed by serological methods.

The serum samples were from patients with a broad spectrum of nematode infections (*Strongyloides stercoralis*, *Ascaris lumbricoides*, filariae, *Trichuris trichiura*, and hookworms), trematode infections (*Schistosoma mansoni*, *S*. *haematobium*, *Paragonimus* spp., *Clonorchis sinensis*, *Opisthorchis felineus*, *Dicrocoelium dendriticum*, and *Fasciola hepatica*); and cestode infections (taeniasis, *Hymenolepis nana*, echinococcosis, and cysticercosis). *Strongyloides* cases with helminth co-infections were excluded. Within the other helminth cases, 15 had more than one helminth infection (S1 Table). In addition, 50 anonymized left-over serum samples from blood donors served as negative controls.

### Serological assays

The study evaluated three commercial kits, *Strongyloides* IgG ELISA (DRG Instruments GmbH, Marburg, Germany), *Strongyloides stercoralis* ELISA (IVD Research Inc., Carlsbad, CA, USA), and *Strongyloides ratti* ELISA (Bordier Affinity Products SA, Crissier, Switzerland). Tests were performed in 2005–2006 in a blinded manner by a senior staff member of the Diagnostic Laboratory, Institute of International Health, following the manufacturer’s instructions. Samples were tested in duplicate. Results were analyzed using mean absorbance values and cut-offs recommended by the manufacturers. The assays represented commonly used tests in Germany and since 2006, none of those three assays has undergone modifications; all are still commercially available (manufacturers´ information, March 2024).

### Statistical analyses

The samples size was chosen in accordance with recommendations for studies on diagnostic tests [14,15].

Sensitivity was defined as the proportion of patients with a positive test result among those with proven infection. Specificity was calculated as the proportion of patients with a negative test result among samples of the control groups. The 95% confidence interval (95% CI) according to Wilson was determined using VassarStats (http://vassarstats.net) and served to compare test performances, assuming significant differences if ranges were not overlapping.

## Results

The analysis included 259 serum samples, 50 from confirmed strongyloidiasis cases, 159 from patients with other helminth infections, and 50 from healthy controls (Table 1). The DRG, IVD, and Bordier assays correctly identified 29, 32, and 28 of 50 strongyloidiasis cases, resulting in sensitivity values of 58%, 64%, and 56%, respectively, with no significant differences (Table 1, Table 2). Specificity values among all 209 non-*Strongyloides* samples for DRG, IVD, and Bordier were 74.2%, 70.8%, and 80.4%, respectively, with a significantly higher specificity for Bordier compared to IVD. Cross-reactivity was frequently observed in the serum panel with other helminth infections and highest in samples with nematode infections (Table 1). The calculated specificities for all samples with non-*Strongyloides* helminthic infections ranged from 62.9% to 76.7%; values for subgroups (nematodes, trematodes, and cestodes) are shown in Table 2. Specificities in the healthy control group were 96.0%, 96.0%, and 92.0%, respectively (Table 2).

**Table 1:** Positive test results of three serological *Strongyloides* assays among cases of helminth infections and healthy controls.

	Samples	*Strongyloides* assay					
	n	DRG		IVD		Bordier	
Positive panel							
* Strongyloides stercoralis*	50	29	(58.0%)	32	(64.0%)	28	(56.0%)
Other nematode infections							
* Ascaris lumbricoides*	26	6	(23.1%)	8	(30.8%)	3	(11.5%)
* *Filariasis	20	16	(80.0%)	16	(80.0%)	12	(60.0%)
* Trichuris trichiura*	26	4	(15.4%)	8	(30.8%)	2	(7.7%)
* *Hookworm	19	10	(52.6%)	10	(52.6%)	3	(15.8%)
* *All (other nematodes)^a^	80	30	(37.5%)	34	(42.5%)	16	(20.0%)
Trematode infections							
* Schistosoma mansoni*	16	2	(12.5%)	2	(12.5%)	1	(6.3%)
* Schistosoma haematobium*	10	0		0		0	
* Paragonimus* spp.	4	3	(75.0%)	3	(75.0%)	2	(50.0%)
* Clonorchis sinensis*	8	2	(25.0%)	4	(50.0%)	1	(12.5%)
* Opisthorchis felineus*	4	3	(75.0%)	3	(75.0%)	3	(75.0%)
* Dicrocoelium dendriticum*	2	1	(50.0%)	1	(50.0%)	0	
* Fasciola hepatica*	3	3	(100%)	3	(100%)	1	(33.3%)
* *All (trematodes)^b^	42	14	(33.3%)	16	(38.1%)	8	(19.0%)
Cestode infections							
* *Taeniasis	12	3	(25.0%)	4	(33.3%)	1	(8.3%)
* *Cysticercosis	10	1	(10.0%)	1	(10.0%)	4	(40.0%)
* *Echinococcosis	10	4	(40.0%)	5	(50.0%)	6	(60.0%)
* Hymenolepis nana*	8	2	(25.0%)	2	(25.0%)	2	(25.0%)
* *All (cestodes)	40	10	(25.0%)	12	(30.0%)	13	(32.5%)
Any helminth infection^a^^-^^c^	209	89	(39.0%)	102	(44.7%)	69	(30.3%)
Healthy controls	50	2	(4.0%)	2	(4.0%)	4	(8.0%)
Total	259	81	(31.3%)	91	(35.1%)	71	(25.1%)

^a^Including 8 cases with ≥1 nematode

^b^Including 5 cases with ≥1 trematode

^c^Including 3 other helminth co-infections (each 1, nematode/trematode, nematode/cestode, and trematode/cestode)

**Table 2:** Sensitivity and specificity of three serological *Strongyloides* assays among samples with helminth infections and healthy controls.

		*Strongyloides* assay					
		DRG		IVD		Bordier	
	n	%	95% CI	%	95% CI	%	95% CI
Sensitivity							
Strongyloidiasis	50	58.0	(43.2–71.5)	64.0	(49.1–76.7)	56.0	(41.3–69.7)
Specificity							
Healthy controls	50	96.0	(85.1–99.3)	96.0	(85.1–99.3)	92.0	(80.0–97.4)
Helminth infections^a^	159	67.3	(59.3–74.4)	62.9	(54.8–70.3)	76.7	(69.2–829)
Nematode infections^a^	80	62.5	(50.9–72.9)	57.5	(46.0–68.3)	80.0	(69.3–87.8)
Trematode infections	42	66.7	(50.4–80.0)	61.9	(45.7–76.0)	80.9	(65.4–90.9)
Cestode infections	40	75.0	(58.5–86.8)	70.0	(53.3–82.9)	67.5	(50.8–80.9)
Total	209	74.2	(67.6–79.8)	70.8	(64.1–76.8)	80.4	(74.2–85.4)

CI, confidence interval

^a^Except strongyloidiasis cases

## Discussion

*Strongyloides* serology offers a diagnostic approach, which is commercially available and less operator-dependent than parasitological methods. Serology is therefore recommended in current guidelines for strongyloidiasis management [1] and in travelers with eosinophilia [16], and as a screening tool in transplant patients [10], before immunosuppressive therapy [17], and in migrants [18]. Different antibody detection techniques have been developed, with ELISA showing the highest sensitivity and specificity [19]. As a major drawback however, such assays lack serological benchmarks, which hampers their evaluation and standardization [20]. As a consequence, studies on serological tests show a multitude of methodological variations and reported performances. A systematic review and meta-analysis from 2020, for example, merged data of 13 serological *Strongyloides* studies, 12 of which evaluated in-house assays [21]. Tests included ELISAs as well as immunofluorescence assays, which were based on different *Strongyloides* species, aimed to detect different immunoglobulin subtypes, and used a broad diversity of reference tests. Sensitivity values ranged from 20% to 100% and the calculated overall sensitivity (72%) is difficult to interpret. A systematic review on strongyloidiasis diagnosis from 2013 included 28 studies on serological assays [22]. The 14 studies on ELISAs based on crude antigen, similar to our study, reported sensitivities from 73% to 100% [22]. Two studies evaluated the commercial assays included in the present study and also used stored serum samples from parasitologically confirmed cases. One study showed a sensitivity of 89% for IVD and of 83% for Bordier [19] and the other 84.2% for IVD [23]. The higher sensitivity compared to our results might be related to differences in the study populations, which were not further characterized in the studies.

Few studies have evaluated *Strongyloides* ELISAs in patients suffering from acute or recent infection in non-endemic countries [24]. The here presented study mainly utilized samples of German travelers and applied a parasitological reference standard (microscopically confirmed strongyloidiasis). Within this group of patients, the three commercial ELISAs showed sensitivities ranging from 56% to 64%. The Bordier assay did not have a higher sensitivity that the DRG kit, which contrasts to a recent Australian study using a serological composite reference standard [25]. These rather low values are in discordance with some of the above mentioned studies, which is most probably explained by our study population with a higher rate of acute or early infections, but might also be related to the heterogeneity of applied methods and reference standards. In populations at risk of helminth polyparasitism, even studies using parasitological gold standards might overestimate the sensitivity of *Strongyloides* serology due to a high background seropositivity. This bias is strongest in endemic areas, but can also affect non-endemic regions, if mainly migrants or refugees from endemic regions are included. A recent retrospective study from France, for example, included 30 proven, probable or possible strongyloidiasis cases, of which 21 were from migrants and nine from travelers; among the latter, only two were microscopically confirmed cases [26]. The authors´ conclusion that the tested assay (*Strongyloides ratti* ELISA, Bordier) was a highly sensitive tool for the diagnosis and screening of travelers seems overly optimistic. Data from 114 parasitologically confirmed cases in Italy showed overall sensitivities of 91% and 90% for the IVD and Bordier ELISAs, respectively. However, 51% samples were non-European migrants; the sensitivity value for the subgroup of European travelers was not provided [27]. Travelers with strongyloidiasis, defined as patients born and residing in a non-endemic country, were analyzed in two reports from reference centers in England [12,28]. In the first study, the Bordier ELISA had a sensitivity of 46% in larvae-positive infections, similar to our results [9]. The second showed that an in-house assay had a sensitivity of 73% in microscopically confirmed cases [28]. Both studies showed a significantly lower sensitivity in traveler compared to migrants, although data on travel duration, allowing a better characterization of the acuity of infections were not provided. The lower sensitivity of *Strongyloides* serology in travelers is probably related to mild infections and early infections, since larvae can appear in stool samples before the production of detectable levels of IgG antibodies in serum [26,29].

Due to shared antigenic fractions, serological cross-reactivity is common among helminths, even across different phyla [30]. This complicates evaluations of serological assays in patients from regions with helminth polyparasitism [28]. Previous works have shown that *Strongyloides* seroassays cross-react with other nematode infections, especially filariasis, affecting their specificity [22]. Cross-reactions to the Trematoda and Cestoda have previously been reported [31], including cystic echinococcosis, which seem to share an antigenic fraction with filarial nematodes [32]. Two recent reviews summarized that the specificities of ELISAs using crude *Strongyloides* antigen showed heterogeneous results, ranging from 29% to 100% [21,22]. *S*. *stercoralis* antigen was less specific than antigen from *S*. *ratti* and *S*. *venezuelensis* [21]. Preincubation with nematode antigens and use of IgG4 have the potential to reduce cross-reactivity and increase specificity [2,22]. However, the latter would diminish sensitivity due to lower IgG4 concentrations [29]; none of these modifications have been commercialized yet. Most studies mention that other nematode infection might cause false positive results; data on cross-reactivity with other helminth phyla have been reported in early studies, but are scarce [33]. In our study, we therefore selected a wide control panel of helminth infections. In accordance with previous studies, the *S*. *ratti-*based Bordier assay was more specific in control samples with other nematode infections (80.0%) than the IVD and DRG assays using *S*. *stercoralis* antigens (57.5% and 62.5%, respectively) [21]. As reported before, filariasis samples showed the highest cross-reactivity [22]. Interestingly, sample cross-reactivity was also observed in trematode and cestode infections, with specificity values ranging from 61.9% to 80.9%. In contrast, all three assays had specificity values of 92.0 to 96.0% in healthy controls from Germany. These data highlight that cross-reactivity might also occur outside the nematode phylum. This phenomenon might be pronounced in travelers presenting early infections, since IgG1, the predominantly produced antibody during this phase, is less specific than later IgG subclasses [29]. Furthermore, IgG-persistence in previously exposed individuals of our control group might have contributed to a diminished specificity. For these reasons, variation of published specificity data might rather reflect on the chosen control populations than on the applied assays, which might explain the contradictory finding of a recent meta-analysis reporting a significantly lower specificity in non-endemic (54%) than in endemic regions (93%) [21]. The limitation of many studies evaluating *Strongyloides* seroassays is that they lack strict inclusion criteria (for sensitivity testing) and sufficient control panels (for specificity testing), resulting in a potential overestimation of test performance [34]. Interestingly, we also observed positivity rates of 4%-8% among the 50 blood donor controls, suggesting possible non-specific reactivity, since donors in Germany are at very low risk of helminth infections and excluded for 6 months to 4 years from donation after visiting tropical regions with any malaria endemicity.

Since the recognition of *Strongyloides* more than 100 years ago, it remains an important goal to improve the diagnostic capacities in the absence of an accepted reference standard [7,12,13]. In endemic regions, disease burden is overestimated by serology, while underestimated by direct methods [34]. Newer studies partly overcome the lack of benchmarks by using Bayesian latent class models [35]. However, *Strongyloides* testing would clearly gain by international efforts including the development of a dedicated target product profile for a serological test, as done for other soil-transmitted nematodes [34]. The presented evaluation in a non-endemic setting, where patients present earlier and have lighter infections than in endemic regions, highlights the limitations of serodiagnosis. In our samples, serology as a sole diagnostic tool would have missed a significant number of cases. To avoid this, some authors recommend empirical treatment with ivermectin or a combination of different serological tests [13]. Importantly, diminished serological responses have also been detected in patients with high parasite loads [12].

In our opinion, healthcare institutions attending travelers and migrants should implement detection methods for *Strongyloides* larvae in stool samples. These are often described as cumbersome and time-consuming [1,21,27]. In our experience, simplifications of the Baermann method, e.g. the Baermann cup technique [36], are rapid, cheap, and easy to perform, and can be implemented in routine microbiological laboratories. Since the sensitivity of serology is diminished in immunocompromised patients [37], the use of direct methods is even more important in this population, which often has high parasite loads. An inverse relationship between high parasite burden and positive serology has also been described in travelers, most probably due to a higher replication rate during early infection [12]. Screening for asymptomatic infection before planned immunosuppression should also not only rely on serology, but include direct methods, possibly molecular tests, or empirical treatment with ivermectin, as suggested by some guidelines [38,39]. Future alternatives include next-generation serological tests based on recombinant antigens or the detection of immune complexes, which might cause false-negative IgG results in chronic infection [13,40]. Coproantigen detection or nucleic acid amplification might also overcome some of the above mentioned obstacles [13]. In resource-poor countries, the implementation of screening techniques for patients at risk for severe manifestations requires the development of affordable and easy-to-perform tests, e.g. IgG detection in urine [41].

Our study has various limitations. Due to the retrospective design using stored serum samples, the study did not represent a real-life population. The applied parasitological gold standard might have caused a bias towards higher parasite loads and more severe clinical manifestations. Due to the study population, very early infections not detectable by serology might have been overrepresented. The control panel with non-*Strongyloides* infections included some cases with helminth co-infections, although all except three were within the same phylum. Since anonymized left-over samples were used, the study did not include detailed demographic and travel-related information as well as clinical data. Although the Baermann technique was routinely applied to patients presenting with gastrointestinal problems or eosinophilia at our institution, individual results of this exam were not available for cases with other helminth infections. Due to lack of a serological gold standard, the control panel might have included cases with previous *Strongyloides* exposure.

In conclusion, commercial *Strongyloides* ELISA kits might contribute valuable information in patients with suspected strongyloidiasis, but due to their limited sensitivity and specificity, they should be interpreted cautiously and used together with parasitological and molecular methods to reduce the risk of missing this chronic and potentially severe infection.

## Supporting information

S1 Checklist STARD checklist for reporting studies of diagnostic accuracy. (PDF)

S1 Table Table with test results of individual samples (1, parasite detected; +, assay with positive result). (XLSX)
